# Activated protein C resistance in Behcet’s disease

**DOI:** 10.1186/1477-9560-11-17

**Published:** 2013-09-02

**Authors:** Hoda Abdel Badaee, Amr Edrees, Sherif Amin, Maher El Amir, Gaafar Ragab

**Affiliations:** 1 Department of Internal Medicine, Cairo University, Cairo, Egypt; 2 Department of Internal Medicine, University of Missouri-Kansas City, Kansas, USA; 3 Department of Clinical Pathology, Cairo University, Cairo, Egypt; 4 Department of Internal Medicine, Fayoum University, Fayoum, Egypt

## Abstract

Behcet’s disease is a chronic multi-system disorder of unknown etiology with protean manifestations. Venous thromboembolism is more common than arterial thrombosis, with deep vein thrombosis being the most frequent. Endothelial dysfunction resulting from vascular inflammation is considered to be an important factor of thrombosis, although the endothelial injury itself cannot completely explain the hypercoagulable state of the disease because other vasculitis syndromes do not increase the risk of thrombosis. The aim of this study is to evaluate the prevalence of activated protein C resistance (APC-R) in Egyptian patients with Behcet’s disease. Also, to detect hyperhomocysteinemia in selected cases (with vascular complications) to assess their relationship with thromboembolic complications. The APC resistance ratio mean in the group of patients with vascular involvement was 2.6 ± 0.8 which was less than the group with no vascular involvement 2.8 ± 0.6, with non- significant P-value (0.5). There was more incidence of ocular lesions in the group of patients with high homocysteine level than the group of patients with normal homocytsteine level with significant P-value (0.08).

## Introduction

Behcet’s disease (BD), initially described by Hulusi Behcet in 1937, is a chronic multi-system disorder of unknown etiology with protean manifestations. The disease is clinically characterized by recurrent aphthous oral ulcers, genital ulcers, ocular inflammation, and skin lesions. It involves a variety of organs including joints, the gastrointestinal tract, the central nervous system, and the vascular system. The main histopathology of BD is vasculitis, and neutrophilic or monocytic vascular inflammation can involve large, medium or small vessels. Thrombotic complications have been reported in approximately 10-40% of BD patients [1].

Venous thromboembolism (VTE) is more common than arterial thrombosis, with deep vein thrombosis being the most frequent [2]. Endothelial dysfunction resulting from vascular inflammation is considered to be an important factor of thrombosis, although the endothelial injury itself cannot completely explain the hypercoagulable state of the disease because other vasculitis syndromes do not increase the risk of thrombosis [3].

Other alterations, such as deficiencies of protein C, protein S and anti thrombin (AT), or the presence of antiphospholipid antibodies and factor V Leiden and prothrombin 20210A mutations, have been considered. Although some studies found an association between these abnormalities and thrombosis in Behcet’s disease [4-8], many other studies did not find any of these abnormalities to be associated with Behcet’s disease [9,10].

Some studies have shown that hyperhomocysteinemia might be assumed to be an independent and correctable risk factor for thrombosis in BD. The association between homocysteine levels and endothelial dysfunction has been shown in patients with BD [11,12].

The aim of this study is to evaluate the prevalence of activated protein C resistance (APC-R) in Egyptian patients with Behcet’s disease. Also, to detect hyperhomocysteinemia in selected cases (with vascular complications) to assess their relationship with thromboembolic complications.

### Patient and methods

The study included thirty two patients with Behcet’s disease. Patients were seen in the outpatient clinic at Cairo University Hospital. All patients fulfilled the International Study Group Criteria for diagnosis of Behcet’s Disease (International Study Group Criteria, 1990) [13]. This necessitates the presence of:

Recurrent oral ulcers observed by physician or patient, which recurred at least three times in one 12-month period, plus two of:

1 Recurrent genital ulcers or scarring observed by physician or patient.

2 Eye lesions: Anterior uveitis, posterior uveitis, or cells in vitreous on slit lamp examination, or retinal vasculitis observed by ophthalmologist.

3 Skin lesions: Erythema nodosum, pseudofolliculitis, or acneiform eruption observed by physician in post-adolescent patients not on corticosteroids treatment.

4 Positive Pathergy test read by physician at 24– 48 hours.

The following patients were excluded from the study:

– Patients under heparin or anticoagulant therapy as these medications affect the result of the test for the APC-R. If the patient was on oral anticoagulant, he should stop (after physician and patient consent) for one week and normalization of prothrombin time.

Patients with prolonged APTT

– Pregnant females

– Women on Contraceptive pills

– Patients have disease or drugs affecting coagulation pathway

– Patients with renal impairment or under treatment with methotrexate, phenytoin, carbamazepine or nitrous oxide were excluded.

Thirty two normal healthy subjects served as control. They have no history of thromboembolism, they did not include pregnant females or women on contraceptive pills.

All patients were subjected to the following:

– Full history and clinical examination.

– Standard laboratory investigations including complete blood picture, liver and kidney function tests.

– Electrocardiogram

– Duplex study for the arterial and /or venous system in patients suspected to have thrombotic event according to the site.

– Patients suspected to have thromboembolic complications, were further investigated by CT chest, ventilation-perfusion scan and echocardiography

– Activated protein C resistance test by coagulation assay for all patients and the control subjects.

– Total plasma homocysteine concentration was measured in ten selected patients from the studied group who had vascular complications.

The test for APC resistance (COATEST) provides an APTT-based assay for semi quantitative determination of the response towards human APC. The prolongation of basal APT clotting time after addition of APC is shorter in plasma from individuals with APC resistance than from individuals with normal response to APC. Plasma is incubated with the APTT reagent for standard period of time. Coagulation is triggered by the addition of CaCl2 in the absence and presence of APC and the time for clot formation is recorded.

Homocysteine was measured by ELISA using Abbott AXSYM system. The normal range in females is 3.36 – 20.44 μmol/L, in males is 5.9- 16.0 μmol/L.

Statistical Package for Social Science (SPSS) program version 9.0 was used for analysis of data. Data was summarized as mean, SD. T test was done for analysis of quantitative data and Non parametric test (Mann Whitney U) was used for analysis of two independent quantitative data when data not symmetrically disturbed. Chi square was used for analysis of qualitative data.

The study was approved by the institution review board of Faculty of Medicine, Cairo University, Egypt and all patients and control subjects signed an informed consent.

## Results

The study included 32 patients with Behcet’s and 32 healthy control persons, 24 patients were males (75%) and 8 patients were females (25%).

The test for activated protein C resistance is considered positive if the ratio ≤ 2. The test was positive in 6 patients (18.8%). The test was positive in 2 control persons (6%). There was non- significant difference in the number of positive test between the two groups, P-value (0.2).

The age of the patients included in the study ranged from 20 to 60 years with a mean 34.8 ± 9.2 years. Duration of the disease ranged from 1 to 24 years with a mean 5.6 ± years. The clinical characteristics of the study patients are summarized in Table 1. The comparison between the clinical characteristics patients with low and normal ratio of APC resistance ratio is summarized in Table 2.

**Table 1:** Clinical characteristics of the study subjects

**Clinical feature**	**N**	**%**
Oral ulcers	32	100
Genital ulcers	30	93.8
Ocular lesions	15	46.9
Vascular involvement	17	53.1
Pathergy reaction	11	34.4
Skin lesions	13	40.6
Cardiac involvement	6	18.8
Pulmonary involvement	10	31.3
CNS involvement	6	18.8
Joint involvement	21	65.6
Gastrointestinal involvement	7	21.9
Low APC r. ratio	6	18.8

*CNS* Central Nervous System.

**Table 2:** Comparison between the clinical characteristics of patients with low and normal ratio of APC resistance ratio

**Clinical feature**	**Low ratio N = 6**		**Low ratio N = 26**		**P-value**
	**N**	**%**	**N**	**%**	
Genital ulcers	6	100	24	92.4	0.7
Ocular lesions	1	16.7	14	53.8	0.2
Vascular involvement	5	83.3	12	46.2	0.1
Pathergy reaction	3	50	8	30.8	0.3
Skin lesions	1	16.7	12	46.2	0.2
Cardiac involvement	2	33.3	4	15.4	0.3
Pulmonary involvement	3	50	7	26.9	0.3
CNS involvement	0	0	6	23.1	0.3
Joint involvement	4	66.7	17	65.4	0.7
GI involvement	2	33.3	5	19.2	0.4

*GI* Gastrointestinal.

Vascular involvement was detected in 17 patients (53.1%) with the following details, the details of vascular involvement is summarized in Table 3.

**Table 3:** Details of vascular involvement in the patients

**Patient number**	**Details**	**Patient number**	**Details**
1	LL DVT	16	Superficial thrombophlebitis
3	DVT (LL, IVC, subclavian)	17	LL DVT, IVC thrombosis, renal vein thrombosis
6	LL DVT, Budd Chiaria	22	Pulmonary artery aneurysm
7	Cerebral infarction	23	LL DVT, Bud Chiari, pulmonary artery aneurysm
8	Pulmonary artery aneurysm	28	Cerebral infarction
10	LL DVT, superior sagittal sinus thrombosis	29	LL and UL DVT
11	LL DVT	30	LL DVT, aortic aneurysm
12	LL DVT, internal jugular vein thrombosis	32	LL DVT, pulmonary artery aneurysm
15	Aortic aneurysm		

*LL* Lower Limb.

*DVT* Deep Venous Thrombosis.

*UL* Upper Limb.

Activated protein C (APC) resistance ratio was done for all patients. Minimum level was 1.4; maximum level was 3.9 with a mean 2.7 ± 0.7, while in the control group the mean was 2.7 ± 0.6 with non-significant probability value (0.9).

Plasma homocysteine level was measured in 10 selected patients with vascular complications. The level was ranging from 8.3 to 25.2 μmol/L with a mean level 14.5 ± 5.6 μmol/L. Three patients had elevated levels (normal range in males 5.9-16 μmol/L and in females 3.36-20.4 μmol/L). The three patients with elevated homocysteine level were males.

The APC resistance ratio mean in the group of patients with vascular involvement was 2.6 ± 0.8 which was less than the group with no vascular involvement 2.8 ± 0.6, with non- significant P-value (0.9).

There were more incidences of ocular lesions in the group of patients with high homocysteine level than the group of patients with normal homocytsteine level with significant P-value (0.08).

The comparison between the clinical characteristics patients normal and high homocysteine level is summarized in Table 4.

**Table 4:** Comparison between the clinical characteristics patients normal and high homocysteine level

**Clinical feature**	**Normal level N = 7**		**High level N = 3**		**P-value**
	**N**	**%**	**N**	**%**	
Ocular lesions	1	14.3	2	66.6	0.08
Pathergy reaction	1	14.3	0	0	0.7
Skin lesions	3	42.9	1	33.3	0.7
Cardiac involvement	2	28.6	2	66.7	0.3
Pulmonary Involvement	4	57.1	1	33.3	0.5
CNS involvement	2	28.6	1	33.3	0.7
Joint involvement	5	71.4	1	33.3	0.3
GI involvement	3	42.9	0	0	0.3

## Discussion

Various mechanisms can contribute to the vascular complications in BD. Although a nonspecific vasculitis involving small and large arteries and veins has been described, the frequency of the thromboembolic phenomena has led to the search for other factors that might enhance intravascular clotting. Studies have investigated the prevalence and role of several factors with procoagulant activity in the thromboembolic phenomena in patients with BD. Most studies investigated these factors separately and yielded conflicting results [9].

This study showed a higher prevalence of activated protein C resistance in patients with Behcet’s disease (18.8%) than in the control group (6%) and was higher in patients with vascular manifestations than those without (29% versus 6.6% respectively) but without statistical significance.

In our study, 6 patients (18.8%) had activated protein C resistance. This frequency is higher than that reported in normal white populations (3 to 7%), and was higher than our studied healthy control (6%). There was no significant difference in the mean APC resistance ratio in our patients and the control group (2.7 ± 0.7 versus 2.7 ± 0.6).

When we compared between the group with vascular complications, the group without and the control group as regard APC resistance, we didn’t find significant difference, however the mean in first group is lower than the other groups. (2.6 ± 0.8, 2.8 ± 0.6, 2.7 ± 0.6 respectively).

Also, we observed that the prevalence of pathergy reaction, cardiac, lung, and GI involvement were higher in the group of APC resistance but again without statistical significance.

Several studies evaluated factor V Leiden or APC resistance as a potential risk factor for vascular complications in BD in different Arab and non-Arab countries. The results were controversial Four studies presented significant association between factor V Leiden mutation (or activated protein C resistance) and vascular complications in BD patients (Gul et al. [2], and Gurgey et al. [14] from Turkey, Mammo et al. [15] from Saudi Arabia, Navarro et al. [16] from Spain), while other studies reported that there was no significant association ( Toydemir et al. [17] from Turkey, Verity et al. [18] from Jordan, Silingardi et al. [19] from Italy).

As many epidemiologic studies supported, mild hyperhomocysteinemia was found to be a risk factor for both venous and arterial thrombosis. Endothelial dysfunction due to inflammation is considered to be an important factor of thrombosis in BD. Homocysteine is reported to enhance endothelial-leukocyte interaction. Some studies have shown that hyperhomocysteinemia might act as independent and correctable risk factor for thrombosis in BD. Moreover, the association between homocysteine levels and endothelial dysfunction has been shown in patients with BD [11].

In our study, we measured plasma homocysteine level in 10 selected patients with vascular complications after exclusion of disease or drugs affecting homocysteine levels. We detected elevated levels in 3 patients (33.3%). Evaluation of association between specific individual clinical manifestations and hyperhomocysteinemia in our study revealed higher prevalence in patients with ocular and cardiac lesions.

There are several studies that evaluated hyperhomocysteinemia in BD patients and their results were conflicting. Aksu et al. [20], detected hyperhomocysteinemia in BD patients with a history of thrombosis.

The significance of hyperhomocysteinemia in ocular behcet’s was evaluated by Okka et al. [21]; they suggested that hyperhomocysteinemia may play a role in ocular involvement of BD.

In conclusion, This study detected a higher prevalence of activated protein C resistance in patients with Behcet’s disease (18.8%) than the control (6%), and was higher in patients with vascular manifestations than those without (29% versus 6.6% respectively) but without statistical significance. Although there was more incidence of positive test for APC resistance in our patients we could not detect statistically significant difference, the number of the patients could be a limiting factor and further studies with higher number of patients are required.

Homocysteine is a risk factor for thromboembolism in BD (30% had hyperhomocysteinemia). Hyperhomocysteinemia is correlated to ocular lesions, but studies with large number of patients will be required to give strong evidence.

The data about hypercoagulablity in BD could have therapeutic implication in the management of deep vein thrombosis and arterial thrombosis in patients with BD. The current recommendations does not suggest the use of anticoagulation for the vascular lesions of BD and suggest the use of immunosuppressive medications only based on the theory that the primary pathology leading to venous thrombosis in BD is the inflammation of vessel wall [22].

## Competing interests

The authors declare that they have no competing interests.

## Authors’ contributions

All authors performed the design, acquisition and validation of data. All authors approved the final version of article.
